# Puerperal Eclampsia

**Published:** 1911-08

**Authors:** L. H. Reeves

**Affiliations:** Decatur, Texas


For Texas Medical Journal.

### Puerperal Eclampsia.

BY L. H. REEVES, M. D., DECATUR, TEXAS.

My reason for presenting this subject is that I consider it one of the gravest conditions with which the obstetrician has to contend. If it comes on before the end of the second stage of labor, two lives are at stake always.

Statistics show that this condition comes once in about every 500 cases—and is more often in primiparas than in multiparas—about 60 per cent of cases occur during labor, 20 per cent before and 20 per cent after labor. Some 25 or 30 per cent of all cases die from toxaemia from suppressed urine, or hemorrhage, or hyperexia.

Many theories have been advanced to explain this condition, most of which are now obsolete. The true cause is yet shrouded in mystery. A prominent theory was that it was toxaemia due to disturbance of metabolism incident to pregnancy. That during pregnancy the eliminating organs of the mother, including the skin, kidneys, respiration and intestinal canal were taxed to double duty, having to eliminate both the excretions of the mother and the child. To supply this the organs were overtaxed.

Post-mortem examinations show pathological changes in both the liver and kidneys. These changes are neither degenerative or inflammatory in character. Some authorities claim that the liver is the site of this condition; that the hepatic cells are overtaxed and are not able to discharge their function and the toxines pass into the blood. The liver and kidneys both being crippled, their resistive power against toxines of any kind is lessened.

It has recently been suggested that this condition is caused by a decreased amount of lime salts in the blood during pregnancy; that many more cases exist where there is no lime in the water used for drinking. It is known that neoplasms and mechanical pressure of the gravid uterus do not cause it.

While we do not know the exact cause, we do know that the blood of the pregnant woman is much more toxic than the blood of a non-pregnant woman. It is loaded with waste matter that can not be eliminated by other channels.

At the present time the most plausible theory of puerperal eclampsia is that the toxines produce changes in the liver and kidneys, and these changes, in turn, increase the already existing toxaemia.

Some of the premonitory symptoms are as follows: First, those which refer to the head—headache. Always think of headache in a puerperal woman as a danger signal—a red light in the distance. Pain may be anywhere in the head. So always investigate when a pregnant woman complains of nervous symptoms. Frequently this symptom is referred to the eyes. She may tell you that she notices peculiar specks and shapes before the eyes when she goes to sleep. Frequently she tells you that she is bilious. She may have no headache at all, or she may tell you that she can see only one side of an object, or can see objects double. Often she complains of ringing in her ears all the time, and she may have total blindness.

*Alimentary Canal.*—The symptoms complained of in reference to the stomach will practically cover the entire field of gastric disturbances. She may complain of any kind of pain, and it may be associated with diarrhoea, or vomiting, or both, good or bad appetite, clean or foul tongue. Vomiting is always an alarming symptom, and we should not be too ready to explain this by something she has eaten some time before. She may be six or nine months pregnant. We should never forget that these disturbances are always suggestive of puerperal eclampsia.

Albuminuria is another symptom that is often exaggerated. Fifty per cent of all pregnant women have albumin in the urine. Crockett says that out of the 50 per cent, 3 to 5 per cent of these have albumin in the urine all the time. You can't depend on the presence or absence of a trace of albumin. However, we should not fail to make a frequent analysis, more especially during the last few months of pregnancy. Yet frequently the danger is done long before albumin is detected in the urine. In fact, some of the most virulent cases never have albuminuria until after they have convulsions. When present, it is nearly always associated with tube casts and blood, if it is abundant. The secretions and excretions are both usually locked up. The pulse is usually rapid with high arterial tension. The patient is often restless and nervous.

In a pratice of nearly ten years I have observed this condition only six times. Five cases were primiparas, one was a multipara and in her eleventh confinement, all her other confinements having been normal. All six cases recovered. In one of the primiparas convulsions came on two weeks before labor, three primiparas had convulsions during labor and two had convulsions after labor. One primipara was delivered of twins, still-born, and another primipara was delivered of a dead child. The other four were delivered of living children. It will probably be interesting to note that two of these cases were sisters, their mother being an epileptic and having died in uremic convulsions. Two others of this family had albuminuria and marked swelling of the limbs and other symptoms of puerperal convulsions, one of which gave birth to a dead child at seven months.

*Prognosis.*—Depends upon: (1) Severity of convulsions; (2) temperature; (3) frequency of convulsions; (4) amount of urine passed; (5) time of pregnancy—the earlier they occur the more fatal.

*Treatment.*—Prophylactic: Your success in treating this condition depends upon the time you detect it, or the likelihood thereof. We should examine a pregnant woman's urine and inquire into her condition frequently and see her from time to time, when we can do so. Yet frequently we are not called and do not even see the lady until she is in labor. This condition of affairs must be overcome by educating the people. Education is the keynote of success in warding off this grave condition. We should take more pains and time to explain to our patients the dangers which may overtake them when in this condition and have them know the absolute necessity of putting themselves under the care of a physician as soon as pregnancy is detected.

One must not depend too much on the test tube and the microscope, as these dangers may develop long before albumin is detected in the urine. When called to see a pregnant woman we should always think of the possibilities of a threatened puerperal eclampsia, and when evidence of such is found we must try to ward it off by prompt elimination of the toxic matter from the woman's body by: (1) Alimentary canal; (2) skin; (3) kidneys.

It has been said that the alimentary canal is the pregnant woman's salvation. Frequently they are constipated. When she complains of nausea and epigastric disturbance, nothing is quite so good as a large dose of calomel (5 to 10 grains), and may add jalap, an equal amount. Sodium phosphate or magnesium sulphate given early in the morning is very good. Empty the large bowel by giving an enema. See that the woman's digestion is kept in good condition; relieve the constipation. Prescribe proper diet; see that the emunctories are going properly and see that she takes the proper amount of exercise. Keep the skin in a healthy condition by proper and frequent bathing.

If the gravity of her condition demands it, put the woman on a chair, wrap a sheet around her and put an alcohol lamp under the chair, and in fifteen or twenty minutes she will perspire freely.

*Kidneys.*—Give plenty of water. If symptoms are very marked, give one-half to one gallon of water in twenty-four hours. It is a good plan to drink a glass every three hours, two glasses when she retires and two glasses when she arises. Sulphur water is good, for she can drink large quantities and not feel full. After symptoms clear up she may take less water. If she refuses to drink so much water, she may take a diuretic, as potassium acetate. However, if there is marked anasarca we should not give so much water, as there is always the danger of oedema of the lungs. Salines by the bowels are always beneficial in this condition.

*Active Treatment.*—We have three points to consider: (1) Control convulsions; (2) promote elimination; (3) deliver the child. To control the seizures, give chloroform while it is on. I do not think chloroform will relieve the convulsions, for you can not get the patient under it quickly enough. To prevent their return, give chloral hydrate by mouth, or preferably per rectum; give morphine and veratrum viride hypodermically. An able colleague of mine tells me that he has used apomorphine in this condition with good results, as it acts as an antispasmodic and relaxes the rigid os. I have never used it in this condition. Watch the heart and hold the pulse down to near 60 or 70. Glonalin is recommended. If there are signs of collapse, use strychnine or whisky. Use hydrotherapy for high temperature. Do not forget the importance of catheterizing the patient and using a saline solution in bowel. If conscious, have patient drink plenty of water and give calomel, gr. X, if she can swallow. If she can not swallow, give croton oil and olive oil on tongue. Another method of elimination is the time honored remedy of blood letting (venesection), used now only in puerperal eclampsia. You may draw off three to ten ounces of blood and inject an equal amount of saline solution. Give mostly milk diet the next few days. May give eggs. *Avoid nitrogenous foods.*

Labor usually comes on promptly after convulsions. But if they cease and the kidneys and other organs seem to do their work properly, do not interfere with gestation. If you have to deliver, secure dilatation and do so as soon as possible.

---

LOEFFLER'S SOLUTION for the local treatment of diphtheria:

| | |
|---|---|
| Menthol | 10.0 grams. |
| Toluol | 26.0 grams. |
| Liq. ferri chloridi | 4.0 c. c. |
| Alcoholis absoluti | 60.0 c. c. |

M. f. mist. Da in vitro nigro.

—*Therapeutic Medicine.*

